# Filtering duplicate reads from 454 pyrosequencing data

**DOI:** 10.1093/bioinformatics/btt047

**Published:** 2013-02-01

**Authors:** Susanne Balzer, Ketil Malde, Markus A. Grohme, Inge Jonassen

**Affiliations:** ^1^Norwegian Marine Data Centre, Institute of Marine Research, P.O. Box 1870, N-5817 Bergen, Norway, ^2^Department of Informatics, University of Bergen, P.O. Box 7803, N-5020 Bergen, Norway, ^3^Department of Molecular Biotechnology and Functional Genomics, University of Applied Sciences Wildau, Bahnhofstraße 1, D-15745 Wildau, Germany and ^4^Computational Biology Unit, Uni Computing, Thormøhlensgate 55, N-5008 Bergen, Norway

## Abstract

**Motivation**: Throughout the recent years, 454 pyrosequencing has emerged as an efficient alternative to traditional Sanger sequencing and is widely used in both *de novo* whole-genome sequencing and metagenomics. Especially the latter application is extremely sensitive to sequencing errors and artificially duplicated reads. Both are common in 454 pyrosequencing and can create a strong bias in the estimation of diversity and composition of a sample. To date, there are several tools that aim to remove both sequencing noise and duplicates. Nevertheless, duplicate removal is often based on nucleotide sequences rather than on the underlying flow values, which contain additional information.

**Results**: With the novel tool JATAC, we present an approach towards a more accurate duplicate removal by analysing flow values directly. Making use of previous findings on 454 flow data characteristics, we combine read clustering with Bayesian distance measures. Finally, we provide a benchmark with an existing algorithm.

**Availability**: JATAC is freely available under the General Public License from http://malde.org/ketil/jatac/.

**Contact**: Ketil.Malde@imr.no

**Supplementary information**: Supplementary data are available at *Bioinformatics* online

## 1 INTRODUCTION

When 454 Life Sciences (now Roche Diagnostics) released the GS20 sequencing platform in 2005 (Margulies *et al.*, 2005), it was the start of a revolution in sequencing technology. It has since been followed by other platforms, both subsequent generations from 454 and competing technologies like Illumina/Solexa and ABI/SOLiD. The increased throughput and decreasing per base cost of these second-generation sequencing technologies have made high-throughput sequencing an affordable tool for many new organisms and applications. The traditional Sanger sequencing is now 30 years old (Sanger *et al.*, 1977), and the error characteristics and artifacts intrinsic to the method are well characterized. Consequently, there are established methods for describing sequence quality (Ewing *et al.*, 1998; Ewing and Green, 1998). Standard methods and tools for detecting and dealing with common contamination like vector sequences or genomic contamination exist, some of them applicable to one or several second-generation sequencing technologies (Chou and Holmes, 2001; Falgueras *et al.*, 2010; Kong, 2011; White *et al.*, 2008). Experienced researchers will also be aware of the risk of artifacts like chimeric sequences arising through different mechanisms (Houseley and Tollervey, 2010; Kanagawa, 2003).

There are numerous approaches to the removal or correction of erroneous sequences or parts of sequences for different applications. These are especially tailored to metagenomics, but also to SNP detection, small RNA discovery and so forth, some of them using 454 pyrosequencing flow data instead of nucleotide sequences, with good results (Huse *et al.*, 2007; Kunin *et al.*, 2009; Quince *et al.*, 2009; Quince *et al.*, 2011; Quinlan *et al.*, 2008; Sogin *et al.*, 2006; Vacic *et al.*, 2008).

### 1.1 Background

Apart from sequencing errors, a second issue accounts for incorrect conclusions in metagenomic studies. Gomez-Alvarez *et al.* (2009) discovered that 454 sequence data contain an over-abundance of reads that are exact or almost-exact duplicates of each other. This comprises both identical reads and reads that start at the same position in the genome but have different lengths or vary slightly, putatively owing to pyrosequencing errors. Although erroneous reads lead to an overestimation of the number of operational taxonomic units in a sample, duplicates artificially inflate the number of reads per operational taxonomic unit, used as an abundance measure. Gomez-Alvarez *et al.* (2009) report between 11% and 35% sequences in metagenomic datasets being artificial duplicates. With the 454 Replicate Filter (Gomez-Alvarez *et al.*, 2009; Teal and Schmidt, 2010), they provide a web-based solution for removing these artifacts, making use of the CD-HIT suite (Li and Godzik, 2006), a fast clustering program for sequences. However, CD-HIT was not specifically designed for 454 pyrosequencing data and operates on fasta input, i.e. on nucleotide sequences rather than on flow data, which is accompanied by information loss (see Section 1.2). With cd-hit-454, Niu *et al.* (2010) provide both a web and a stand-alone tool for the removal of artificial duplicates in metagenomic pyrosequencing data. Also, PyroCleaner (Mariette *et al.*, 2011) has been specifically designed for 454 data, but all these tools work on nucleotide sequences. Our main motivation for developing JATAC was to aid metagenomic projects in the tradition of 454 Replicate Filter and cd-hit-454, but leveraging additional information present in flow data. JATAC targets both the assembly of (meta)genomes and the accurate estimation of community compositions. Gomez-Alvarez *et al.* have shown that failure to remove duplicates resulted in misleading conclusions on the gene space in soil metagenomes (Gomez-Alvarez *et al.*, 2009). Furthermore, methods using sequence coverage to identify repeats (e.g. Malde *et al.*, 2006; Phillippy *et al.*, 2008) should not be applied to pyrosequencing data without first filtering duplicates.

### 1.2 Nucleotide space versus flow space

In 454 pyrosequencing, around one million DNA molecules are sequenced in parallel (∼100 000 in the benchtop solution GS Junior), generating a series of so-called flow values for each molecule. One flow value corresponds to the number of identical bases incorporated in a single flow. The cycling order of the nucleotides is maintained throughout the sequencing process (T, A, C, G representing one flow cycle). The underlying sequence is inferred from the respective flow values of each nucleotide.

Flow values refer to the signal strength of the sequencing reaction (for details on the sequencing chemistry, see Margulies *et al.*, 2005). With increasing homopolymer length, the signal differences and thereby the discriminatory power of the base calling decrease, resulting in a well-known uncertainty about exact homopolymer lengths, especially for long homopolymers (Gilles *et al.*, 2011; Huse *et al.*, 2007; Margulies *et al.*, 2005). As nucleotide homopolymer length can only be expressed in integers, it is indispensable to carry out analyses based on flow data (expressed as double decimal values) instead of nucleotide sequences, i.e. in ‘flow space’ instead of ‘nucleotide space’.

The native output format of 454 pyrosequencing is the binary standard flowgram format (*.sff). It contains the flowgram for each read, whereby each flowgram consists of a sequence of flow values representing base incorporations. One flowgram corresponds to 800 flows (200 flow cycles) in the GS FLX/Junior Titanium chemistry, i.e. one flow value per position 1-800. The GS FLX+ chemistry uses 1600 flows (400 flow cycles).

In the following, we present a reference-free method and algorithm named JATAC that identifies duplicate reads based on the flowgram. Methods operating in flow space have been shown to be superior to methods working in nucleotide space, e.g. for noise removal in metagenomics amplicon data (see earlier in the text). Our results indicate that this is also the case for duplicate removal.

## 2 DUPLICATE FILTERING

### 2.1 Natural versus artificial duplicates

Library generation for 454 pyrosequencing involves an emulsion polymerase chain reaction (PCR) step where water-oil droplets are formed (Tawfik and Griffiths, 1998; Williams *et al.*, 2006). This segregates the complex reaction mixture into miniaturized compartments and allows for highly multiplexed DNA amplification reactions. In these so-called micro-reactors, single DNA molecules are clonally amplified onto beads and are then deposited on a PicoTiterPlateTM (PTP) for sequencing (Leamon *et al.*, 2003; Margulies *et al.*, 2005). An inherent artifact of 454 library preparation and sequencing is the generation of artificial duplicate sequences as a result of the emulsion PCR step.

There are three suspected sources for artificial duplicates: Emulsion PCR, background amplicon contamination and signal cross-talk on the PTP sequencing device.

Usually, the low DNA-to-bead ratio minimizes the possibility of loading a single bead with two distinct DNA molecules, thereby generating mostly single-copy beads for sequencing (Zheng *et al.*, 2010). Conversely, many beads will remain empty, and droplets containing several beads and a single DNA molecule will therefore result in loading these beads with identical copies of the original DNA molecule. The strongest manifestation of overloading empty beads with identical molecules can be observed during unwanted emulsion breakage, when the emulsions become chemically unstable during thermal cycling and the micro-reactors fuse into larger droplets.

An amplicon contamination of amplified library DNA molecules from a previous sequencing run can also lead to duplicate reads in following runs, but these types of duplicate errors can normally be avoided by preventing cross-contamination of sequencing library samples.

Signal duplicates are an effect of well-to-well cross-talk, where strong signals ‘bleed’ into neighbouring empty wells (Briggs *et al.*, 2007). With the launch of the 454 Titanium chemistry, well cross-talk has been minimized by metal coating of the PTP well surface (Roche Applied Science, 2008).

Most likely, the main source of duplicates can be attributed to the emulsion PCR step. As the beads are randomly distributed on the plate, and the DNA on each bead is amplified and sequenced independently, the final length and error content of the sequence read can differ, but in all cases, the starting position of the read will be identical for all duplicates.

In contrast to artificial duplicates, duplicates can also arise ‘naturally’, i.e. by chance through sampling DNA molecules that start at identical positions or in repetitive regions of a genome. For genomic shotgun sequencing projects, there is a correlation between genome coverage and the percentage of natural duplicates. With increasing read density, the amount of natural duplicates will also increase. In metagenomic datasets of high complexity, i.e. in the absence of dominant species, the percentage of natural duplicates should be very low. For metatranscriptomic samples, the discrimination of natural and artificial duplicates is much more difficult, as some highly expressed RNAs will be sequenced much more often. For such datasets, it is challenging to distinguish between artificial and natural duplicates (Niu *et al.*, 2010).

### 2.2 Benchmark dataset construction

To compare the performance of JATAC and cd-hit-454, we generated three benchmark datasets, each consisting of a dataset of (real) reads and information about duplicates within each set of reads. We chose sequence datasets where a reference was available to accurately assess duplicate removal. Benchmarking on reference-free metagenome datasets would have resulted in a set of duplicate clusters and an expected duplication rate but would give no indication of the accuracy of each method for duplicate detection.

We used the GS Reference Mapper v. 2.6 (Roche Applied Science, 2008) with default settings and processed the results from the benchmark datasets in the following way: to precisely get the correct alignment for the beginning of each read, we independently mapped our data to the original and reverse complement genome. The BAM file generated by the mapper was converted into SAM format using samtools (Li *et al.*, 2009) and split into matches to the forward and reverse strands of the genome, retaining only forward matches relative to the respective reference (original/reverse complement). A subset of alignments was identified by extracting only unique alignment start positions and 16-nucleotide sequence prefixes, discarding alignments where the initial part of the read was masked (i.e. having ‘H’ as the first element of the field). Clusters of duplicate alignments were then extracted by grouping all reads with the same prefix and aligned position. This procedure is for reference dataset generation only and not to be confused with the JATAC algorithm (see Section 2.3).

**Fig. 1.**
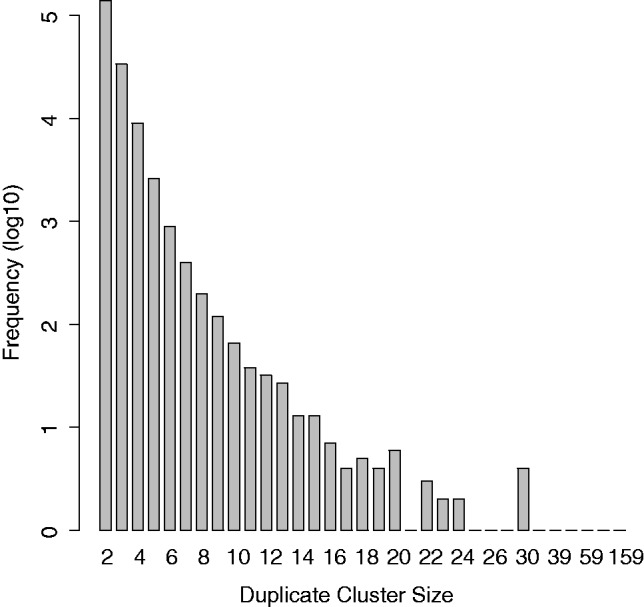
True duplicate cluster sizes from *D.labrax* benchmark dataset. The biggest cluster contains 159 reads (see Fig. 2)

**Fig. 2.**
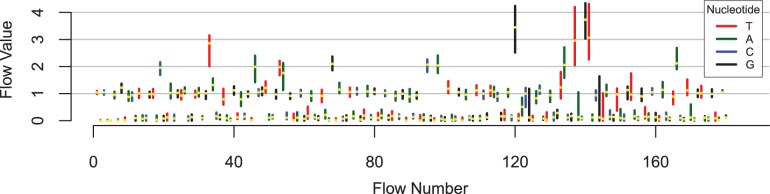
Biggest flowgram cluster from *D.labrax* reference dataset (159 reads). Each vertical bar represents the range of flow values in this flow. The median flow value is plotted in yellow. The wide range of flow values in longer homopolymers, as well as the broad distributions of flow values at flow 122-124 and 144-145 represent under- and overcalls leading to indels and substitutions in the resulting nucleotide sequences. The longest flowgram was trimmed after flow no. 180 by the 454 software. The reads in the cluster have an average length of 88 bp in nucleotide space (+/− 14 bp, maximum 102 bp)

For the first benchmark dataset, we mapped 1 270 325 *Dicentrarchus labrax* (sea bass) 454 GS FLX Titanium reads to the corresponding (Sanger-sequenced) reference scaffold (Kuhl *et al.*, 2010). As a result, 35.80% of the 1 270 325 reads are part of a cluster of at least two flowgrams that map to the same position in the reference genome. By subtracting one representative per duplicate cluster, we estimated the overall duplicate rate for *D.labrax* to be 20.18%. Of all duplicate clusters, 75% contain two, another 18% contain three and 5% contain four flowgrams. The biggest cluster contains 159 flowgrams (see Figs 1 and 2). The genomic reference used for sea bass is incomplete leading to a possible over-estimation of artificial duplicates. However, this does not introduce any bias in favour of any of the clustering algorithms. In other respects, this dataset is ideal as a benchmark, as the 454 sequences stem from the same individual on which the reference is based while the reference was constructed using a separate sequence set.

The second and third benchmark dataset consisted of two 454 GS Junior Titanium runs of an isolate of *Escherichia coli* O104:H4, containing 137 528 and 135 992 reads, respectively. This Shiga toxin producing strain was responsible for an outbreak of food poisoning in Germany in 2011 (Loman *et al.*, 2012).

### 2.3 Removal of duplicates with JATAC

We cluster flowgrams rather than reads and operate solely in flow space (see Section 1.2). We take into account the 454 key and quality trimming information included in the flow data files, which means that only informative flow values are used in the duplicate removal algorithm [see Equation (3)].

#### 2.3.1 Preclustering

Our clustering algorithm involves calculating the pairwise distances of all flowgrams. As this is computationally expensive on a dataset with more than a million flowgrams (typical 454 FLX Titanium run), we perform a preclustering step that creates subsets of flowgrams. Subsequent clustering is only performed on these subsets, which means that flowgrams from different subsets cannot be identified as duplicates of each other.

For preclustering, we use a varying seed of at least eight flows, starting with the first flow. For each of these flows, we only take into account if the flow value was ‘negative’ (i.e. 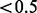) or ‘positive’ (i.e. 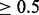, leading to at least one called base). For preclusters containing >2000 flowgrams, we gradually increase this seed to further split them up. In addition, we require flowgrams within one precluster to start with the same homopolymer length.

#### 2.3.2 Distance measures

**Fig. 3.**
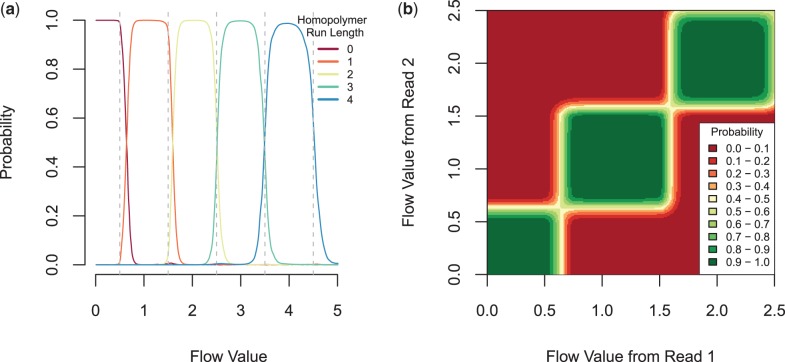
(**a**) Probability for homopolymer lengths given a flow value [see Equation (1)]. (**b**) Probability for two homopolymer lengths being equal, given two flow values [see Equation (2)]. Both figures show the probabilities related to the first 10 flow cycles; for details, see Balzer *et al.* (2010)

To assess how similar two flowgrams are, we define a distance measure. This is similar to the distance definition by Quince *et al.* (2011) but directly compares two flowgrams rather than one flowgram with a perfect flowgram consisting of integers. We begin by applying Bayes’ Theorem to calculate the probability for a homopolymer length being equal to h when observing a flow value f (see Fig. 3a):
(1)
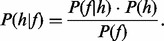

The prior—the homopolymer length distribution *P*(*h*), the flow value distribution *P*(*f*) and the likelihood distribution 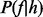 are taken from earlier analyses and consist of an average smoothed distribution of *D.labrax* and *E.coli* flowgrams, mapped to their respective reference genomes and taking into account quality degradation towards later flow cycles. Determination of these distributions has been described in detail in Balzer *et al.* (2010). We argued earlier that the distributions are representative for other species for homopolymer lengths up to 5, and they can be downloaded from the flower website (http://biohaskell.org/Applications/Flower). Furthermore, we excluded any overfitting issues by demonstrating that the probability lookup tables are more or less interchangeable without impacting the outcome too much: when clustering *D.labrax* data with the use of a lookup table created from *E.coli* flow value distributions, our results were equally good as when using the smoothed average distribution from *D.labrax* and *E.coli* (see Section 2.3.2).

If we assume that two flowgrams, *fg_a_* and *fg_b_*, are independent from each other, then we can further calculate the probability that the homopolymer lengths, *h_ai_* and *h_bi_*, are equal, given two flow values, *f_ai_* and *f_bi_* (see Fig. 3b), the latter being flow values from *fg_a_* and *fg_b_* in the same flow (i.e. position) *i*.
(2)
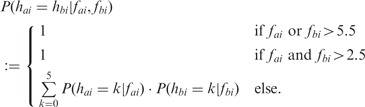



For reasons of algorithm robustness, we assign a fixed probability score of 1 if at least one flow value is >5.5 or if both flow values are >2.5, thereby giving lower and better resolved flow values more weight in similarity calculations [see Equation (3)]. The latter corresponds to the observation that the most common sequencing error in 454 pyrosequencing is due to incorrectly determined homopolymer stretches (see Section 1.2).

In all other cases, we sum up the probabilities for the two flow values leading to the same homopolymer length 0, … ,5 to obtain a realistic estimate for the two values resulting in homopolymers of equal length. The flow-position-wise calculation of probabilities ensures that the two flow values in question always relate to the same nucleotide (see Fig. 2).

It is assumed that the flow values of one flowgram are not correlated. The assumption is strictly speaking invalid owing to the occurrence of carry forward and incomplete extension, phenomena that the 454 software partly corrects for. Under this assumption, we can define the distance 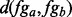 between two flowgrams as follows:
(3)
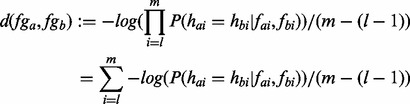

with


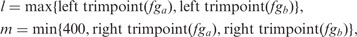


the trimpoints being defined by the 454 software.

#### 2.3.3 Hierarchical flowgram clustering

Once we have defined our distance measure, we iterate through the files that contain the preclustered flowgrams (see Section 2.3.1) and perform agglomerative clustering on one file at a time.

We now start with one flowgram per cluster (i.e. each cluster being a singleton) and calculate all pairwise distances between flowgrams. In each clustering step, the two clusters, which have the smallest distance from each other, are combined into a new cluster. Two updates are then performed: First, a consensus flowgram is determined for the new cluster by calculating the per-flow median of flow values from all flowgrams in this cluster (quality-trimmed regions only). Second, the distances between the new cluster and all other clusters are updated. We continue clustering until all pairwise distances between clusters exceed a given stringency threshold.

We experimented with different threshold settings for the distance measure. Also, we only use the first 400 flow values of a flowgram [or all flow values up to the lowest trimpoint, see Equation (3)].

Our method of calculating a consensus flowgram is based on our observation that flow values in true duplicate clusters tend to stretch out to one side of the integer for each flow position (see Fig. 2). Correspondingly, we calculate the median flow value per flow.

#### 2.3.4 Output

We have implemented three modes for determining a representative of a flowgram cluster: ‘longest’, ‘best’ or ‘consensus’. Also, we provide both fasta and sff output to meet the needs of a broad range of users. Choosing the longest read from a cluster is straightforward; choosing the best read involves calculating the squared sum of the flow values’ distance to the corresponding integers, normalized by flowgram length. Obviously, flow values that lie close to integers have a high accuracy. The consensus flowgram is the median flowgram that previously has been used to (re-)calculate the distances between clusters in the clustering algorithm. When using the consensus option, the output of a cluster is therefore an artificial consensus flowgram of all flowgrams in the cluster (at least if a cluster contains more than one read).

### 2.4 Benchmark of methods

In general, when calculating the duplicate rate for a dataset without comparing with a reference, the result strongly depends on the stringency at which reads are regarded as being ‘similar enough’. We ran JATAC on all *D.labrax* FLX Titanium and *E.coli* Junior Titanium reads (see Section 2.2) and clustered them at different stringency thresholds, the threshold being the maximum allowed distance when combining two clusters [see Equation (3)]. Also, we used the command line version of cd-hit-454 (v. 4.6, Li and Godzik, 2006; Niu *et al.*, 2010) to cluster our shotgun data at different stringency settings (between 91% and 100%), where 98% is the default stringency in cd-hit-454. Results are given in Table 1.

**Table 1.** Duplicate clustering results for cd-hit-454 and JATAC

Stringency^a^	Estimated duplicate rate/Jaccard index		
	*E.coli* (Run 1)	*E.coli* (Run 2)	*D.labrax*
cd-hit-454			
100%	3.24%/0.30	6.56%/0.29	2.73%/0.09
99%	8.20%/0.75	15.64%/0.73	13.21%/0.45
98%	9.29%/0.82	17.59%/0.81	19.13%/0.64
97%	9.57%/0.83	18.04%/0.82	20.82%/0.66
96%	9.67%/0.83	18.18%/0.82	21.35%/0.65
95%	9.72%/0.83	18.25%/0.83	21.58%/0.63
94%	9.74%/0.83	18.29%/0.83	21.72%/0.61
93%	9.76%/0.83	18.30%/0.83	21.81%/0.59
92%	9.77%/0.83	18.31%/0.82	21.88%/0.59
91%	9.77%/0.83	18.32%/0.82	21.88%/0.59
JATAC			
0.00	0.00%/0.00	0.00%/0.00	0.00%/0.00
0.01	7.66%/0.71	15.10%/0.72	18.28%/0.65
0.02	8.60%/0.78	16.67%/0.79	20.40%/0.72
0.03	9.11%/0.82	17.54%/0.83	21.36%/0.74
0.04	9.41%/0.84	18.05%/0.85	21.89%/0.75
0.05	9.63%/0.85	18.41%/0.86	22.22%/0.76
0.06	9.77%/0.86	18.65%/0.86	22.45%/0.77
0.07	9.89%/0.86	18.82%/0.87	22.61%/0.77
0.08	9.97%/0.86	18.96%/0.87	22.75%/0.77
0.09	10.03%/0.87	19.08%/0.88	22.85%/0.77
0.1	10.08%/0.87	19.16%/0.88	22.93%/0.77
True duplicate rate	9.65%	18.61%	20.18%

^a^The clustering stringency corresponds to a sequence identity threshold for cd-hit-454 and to a distance threshold for JATAC. For the latter, a higher distance corresponds to lower identity.

**Fig. 4.**
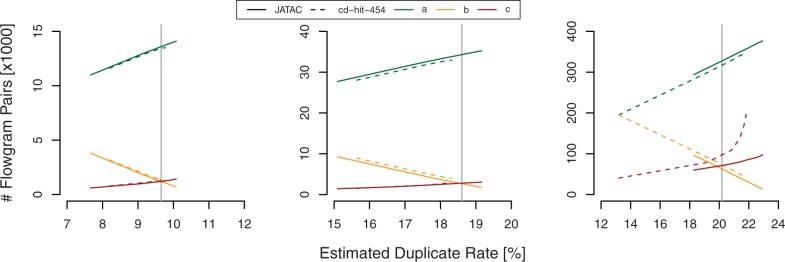
Comparison of JATAC and cd-hit-454 duplicate clustering at different stringency settings and estimated duplicate rates surrounding the true duplicate rate (vertical grey line). The range of parametrization lies between 0.02 and 0.10 (distance threshold) for JATAC and between 99% and 92% for cd-hit-454 (identity threshold). Left: *E.coli* (Run 1). Centre: *E.coli* (Run 2). Right: *D.labrax*. For explanation of a, b and c pairs, see the text

To evaluate to what extent our JATAC algorithm allows for a more effective removal of artificial duplicates compared with the nucleotide sequence-based cd-hit-454, we need a measure that compares two sets of clusters. The Jaccard index
(4)
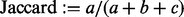



can be used to compute the degree of similarity between the real set of true duplicate clusters (from our reference, see Section 2.2) and the set of duplicate clusters identified by the respective clustering algorithm. Those flowgram pairs that are correctly identified as duplicates of each other are counted as *a*; those that are not identified as duplicates, although they map to the same position in the reference genome, are counted as *b*; and those that are incorrectly identified as duplicates are counted as *c* (see Fig. 4). The flowgram pairs *b* and *c* can vaguely be understood as false positives and false negatives from a classification problem. However, the calculation of common classification indicators such as sensitivity and specificity would be misleading here, as it is not sufficient to identify a flowgram as an artificial duplicate of *some* other flowgram, but it is relevant *which* flowgrams are clustered together.

JATAC outperformed cd-hit-454 on all three datasets, regardless of sequencing platform (GS FLX/Junior Titanium), actual duplication rate or complexity (see Table 1 and Fig. 4) at similar estimated duplicate rates. We have experienced that a slight overestimation of the true duplicate rate gives the best results in terms of Jaccard index. This is true for both JATAC and cd-hit-454.

For the second *E.coli* dataset, cd-hit-454 underestimated the true duplicate rate even at a similarity threshold of 90% (data not shown). This illustrates one caveat when using duplicate removal tools such as JATAC or cd-hit-454, namely to determine at which stringency the reads should be filtered. However, the cd-hit-454 identity threshold and the JATAC distance threshold are not directly comparable. A JATAC distance of 0 does not exactly correspond to a cd-hit-454 stringency of 100%, as it is a lot more probable that two artificial duplicates share the same nucleotide sequence than that they share the exactly identical flowgram to the second decimal place. We have found that a distance measure of 0.05 is a good starting point for duplicate analyses resulting in a reasonable Jaccard index.

Additionally, we tested the effect of duplicate removal on assembly performance of the *E.coli* genome. Therefore, the two datasets were independently filtered for duplicates (keeping the longest read per cluster) and assembled together using Newbler. The rationale behind this was to reduce assembly artifacts from low coverage. In addition, owing to the separate duplicate filtering, we only removed a minimal amount of natural duplicates. We scored the resulting assemblies for a limited parameter set using Mauve assembly metrics (Darling *et al.*, 2011) and found no striking differences between JATAC and cd-hit-454 filtered assemblies. For both tools, the N50 increased to 126 844 bp in comparison with the unfiltered assembly with an N50 of 106 414 bp (see Supplementary Material). We conclude that the high and identical N50 value obtained using both approaches is likely to represent the highest possible assembly continuity for the given dataset and read length (Cahill *et al.*, 2010).

## 3 DISCUSSION

In this article, we have quantified the room for improvements when filtering 454 pyrosequencing shotgun data for artificial duplicates. We have successfully shown that, by the use of 454 flow data, a higher rate of artificial duplicates can be identified than by using sequence data only. Artificially duplicated reads can—apart from a generally higher processing and memory requirement—lead for example to incorrect conclusions about metagenomic dataset composition (Gomez-Alvarez *et al.*, 2009) or to biased quantification in digital karyotyping experiments (Dong *et al.*, 2011). Another likely problem could be false positive single nucleotide polymorphism calls in the presence of duplicated erroneous sequences. However, too stringent filtering might lead to an underestimation of abundance (Niu *et al.*, 2010).

Both JATAC and cd-hit-454 cannot distinguish natural from artificial duplicates, but the percentage of natural duplicates can be estimated from sequencing coverage by calculating the probability of multiple reads randomly starting at the same position (Niu *et al.*, 2010).

Although cd-hit-454’s estimated duplicate rates were comparable with JATAC’s estimations, the calculated cluster composition at similar duplication rates was of lower quality, manifested in a lower Jaccard index. This is likely the result of JATAC being better at handling homopolymer discrepancies and taking flow order into account, whereas cd-hit-454 is operating mostly on global similarity scores. The distance calculation in JATAC is a more robust way of finding duplicates, as it first identifies read pairs with different homopolymer lengths at low distances. Only with higher distance thresholds, reads with substitutions are taken into account. This behaviour closely models the 454 sequencing chemistry where substitution errors are less common than indels. Interestingly, the Jaccard index calculated from running cd-hit-454 on the *D.labrax* dataset degraded much faster around the true duplicate rate when compared with JATAC. This degradation could not be observed in the bacterial datasets and is likely due to a higher probability of matching unrelated sequences from a complex background. This phenomenon could also be relevant to metagenomic experiments of highly diverse communities, where tools such as cd-hit-454 and JATAC are most useful. A comprehensive overview of applications and effects of duplicate filtering, e.g. on genome assembly, can be found in Li *et al.* (2012).

JATAC’s improved duplicate identification comes at a computational price, and its speed depends on the number of reads and the degree of duplication. JATAC takes up to several hours to filter an sff file for duplicates, ∼1.5 h for a typical GS Junior run.

We have also evaluated JATAC on IonTorrent flow data, as both platforms share the same data format (sff). Although it is in principle possible to analyse ionograms using JATAC, the underlying flow data model has been optimized for pyrosequencing data, which is why we do not recommend JATAC for IonTorrent data in its present version.

## Supplementary Material

Supplementary Data
