# Supplementary material for: Filtering duplicate reads from 454 pyrosequencing data
Source: Bioinformatics. 2013 Feb 1;29(7):830–6. doi: 10.1093/bioinformatics/btt047 (PMC3605598; doi:10.1093/bioinformatics/btt047)
Supplement: Supplementary Data [file supp_29_7_830__index.html]

Filtering duplicate reads from 454 pyrosequencing data — Filtering duplicate reads from 454 pyrosequencing data — Filtering duplicate reads from 454 pyrosequencing data — Supplementary Data 

# Filtering duplicate reads from 454 pyrosequencing data

## Supplementary Data

files

**Files in this Data Supplement:**

- Supplementary Data - xls file
